# Genes involved in pulmonary hypertension of mice with endothelial-specific ablation of guanylyl cyclase A

**DOI:** 10.1186/2050-6511-14-S1-P77

**Published:** 2013-08-29

**Authors:** Franziska Werner, Katharina Völker, Claus-Jürgen Scholz, Margarethe Goebel, Michael Seimetz, Baktybek Kojonazarov, Kai Schuh, Bhola K Dahal, Ralph T Schermuly, Heike Oberwinkler, Birgit Gaßner, Michaela Kuhn

**Affiliations:** 1 Institute of Physiology, University of Würzburg, Germany; 2 ZKF Würzburg, University of Würzburg, Germany; 3 Department of Internal Medicine, University of Giessen, Germany

## Background

Pulmonary arterial hypertension (PAH) is a rare progressive, usually fatal lung disease of different ethiologies. Endothelial cell dysfunction contributes to the pathogenesis, although the specific mechanisms are not clear. Impaired vascular cGMP signaling seems to be involved in the development of pulmonary vascular remodeling and constriction, because drugs preventing cGMP degradation or stimulating cGMP production showed beneficial effects. Atrial (ANP) and B-type natriuretic peptides (BNP) both act through the guanylyl cyclase-A (GC-A) receptor and stimulate cGMP production in all vascular cell types (i.e. endothelial cells (EC), smooth muscle cells (SMC) and fibroblasts). The purpose of the study was to analyze whether mice with global or conditional, EC- or SMC-restricted GC-A ablation develop PH under normoxic conditions and which genes are differentially expressed.

## Results

Mice with global or conditional, EC-restricted GC-A deletion develop PH even under normoxic conditions. Right ventricular (RV) systolic pressure is increased.This is accompanied by RV hypertrophy and dysfunction, enhanced muscularization of small pulmonary arteries and arteriolar rarefaction. At difference, deletion of GC-A in SMC did not result in PH. Because mice with EC-restricted ablation of GC-A (EC GC-A KO) develop PH, a microarray analysis was performed to identify differentially expressed genes. Precapillary pulmonary arteries (21-87 µm diameter) were dissected by Laser microdissection, RNA was isolated and transcribed into cDNA and applied to Mouse 430_2 Affimetrix chips. The mRNA expression of 34 genes was up- and of 13 genes was downregulated in precapillary pulmonary arteries of EC-GC-A-KO mice. The top ten overexpressed mRNAs were Epac2 (3.6 times), adenosine deaminase, tRNA-specific 2 (3.1 times), UBX domain protein 7 (3.0 times), ring finger protein 128 (2.8 times), DAZ interacting protein 3 zinc finger (2.8 times), UDP-GlcNAc:betaGal beta-1,3-N-acetylglucosaminyltransferase 5 (2.7 times), LEM domain containing 3 (2.6 times), retinol dehydrogenase 10 (2.2 times), glypican 4 (2.2 times) and splicing factor proline/glutamine rich (2.2 times). The top ten downregulated mRNAs were charged multivesicular body protein 4C (-3.2 times), family with sequence similarity 199, X-linked (-3.1 times), transient receptor potential cation channel, subfamily M, member 7 (-2.8 times), pecanex homolog (Drosophila) (-2.7 times), serine/arginine-rich splicing factor 18 (-2.6 times), zinc finger and BTB domain containing 1 (-2.5 times), importin 5 (-2.4 times), adenomatosis polyposis coli (-2.3 times), myogenic factor 5 (-2.3 times) and solute carrier family 35 member B3 (-2.2 times).

## Conclusion

These observations illustrate that ANP/GC-A/cGMP signaling critically affects pulmonary EC proliferation/viability and the paracrine crosstalk of pulmonary EC with SMC. Furthermore, the pulmonary GC-A/cGMP responsiveness to ANP was mitigated in PAH patients. Here, we provide new mechanistic insights into PAH and genes involved in its development, i.e. the role of altered endothelial natriuretic peptide signaling.

